# p38 MAPK, microglial signaling, and neuropathic pain

**DOI:** 10.1186/1744-8069-3-33

**Published:** 2007-11-01

**Authors:** Ru-Rong Ji, Marc R Suter

**Affiliations:** 1 Pain Research Center, Department of Anesthesiology, Brigham and Women's Hospital and Harvard Medical School, Boston, Massachusetts, USA

## Abstract

Accumulating evidence over last several years indicates an important role of microglial cells in the pathogenesis of neuropathic pain. Signal transduction in microglia under chronic pain states has begun to be revealed. We will review the evidence that p38 MAPK is activated in spinal microglia after nerve injury and contributes importantly to neuropathic pain development and maintenance. We will discuss the upstream mechanisms causing p38 activation in spinal microglia after nerve injury. We will also discuss the downstream mechanisms by which p38 produces inflammatory mediators. Taken together, current data suggest that p38 plays a critical role in microglial signaling under neuropathic pain conditions and represents a valuable therapeutic target for neuropathic pain management.

## Background

Injuries of the nervous system, including peripheral nervous system (PNS, e.g. peripheral nerves, dorsal root ganglia, and dorsal roots) and central nervous system (CNS, e.g. spinal cord and thalamus), often result in neuropathic pain. These injuries may result from diabetic neuropathy, viral infection, major surgeries (e.g. amputation, thoracotomy), spinal cord injury, and stroke [1-3]. Spontaneous pain, described as shooting, lancinating or burning pain, and mechanical allodynia (painful responses to normally innocuous tactile stimuli) are distinct symptoms of neuropathic pain, although neuropathic pain is also characterized by heat hyperalgesia, mechanical hyperalgesia, and cold allodynia. Neuropathic pain is a consequence of neural plasticity, developed both in the PNS (peripheral sensitization) and CNS (central sensitization). After nerve injury, neuropathic pain can arise from injury discharge at the site of axonal injury and ectopic/spontaneous activity in dorsal root ganglion (DRG) neurons [4-6]. Inflammatory mediators (e.g. cytokines) play a critical role in the generation of spontaneous activity and neuropathic pain. Peripheral nerve injury also induces marked phenotypic changes in DRG neurons [1,2]. While spontaneous activity from primary afferents drives central sensitization, central sensitization is responsible for persistent neuropathic pain. Central sensitization may also directly drive neuropathic pain in central neuropathic pain conditions caused by spinal cord injury or stroke. Central sensitization is induced by enhanced synaptic strength in the spinal cord and brain regions, due to an increase in excitatory synaptic transmission (e.g. AMPA and NMDA currents) or/and a reduction in inhibitory synaptic transmission (e.g. GABA currents) [7-9]. In addition to increased primary afferent input, enhanced descending facilitation also contributes to spinal neuron hypersensitivity and neuropathic pain [10,11].

Despite our intensive investigation on neuronal mechanisms of neuropathic pain, current treatment has only resulted in limited success. Most analgesics are designed to block neurotransmission, but pain rapidly comes back after drug effects wear off. This is because many "inflammatory mediators (IFMs)" are still produced to activate nociceptive neurons in the PNS and CNS, causing pain hypersensitivity. These IFMs include proinflammatory cytokines [interleukin-1beta (IL-1β), interleukin-6 (IL-6), and tumor necrosis factor-alpha (TNFα)], prostaglandin E_2 _(PGE_2_), nitric oxide, nerve growth factor, etc. Unlike neurotransmitters, these IFMs are mainly produced by non-neuronal cells. The IFMs are produced not only at the site of nerve injury by Schwann cells, keratinocytes and immune cells, but also by glial cells in the spinal cord. Microglia are regarded as a main source of IFMs in the CNS [12,13]. Although brain-derived neurotrophic factor (BDNF) is not regarded as an IFM, it is produced by microglia and plays important role in neuropathic pain development [14]. Thus our list of IFMs also includes BDNF. Since IFMs produced by spinal microglia are crucial to the development of central sensitization and neuropathic pain (see below), it is extremely important to know how these IFMs are produced in microglia. We will summarize the data showing that p38 MAPK is a key regulator of IFM synthesis and release in microglia and also an essential contributor to neuropathic pain sensitization.

### Microglia and neuropathic pain

Although glial cells were originally regarded as supporting cells in the CNS, mounting evidence indicates that glia actively communicate with neurons and contribute importantly to the development of different types of neurodegenerative diseases. Increasing evidence also suggests that glial cells in the spinal cord play an important role in pain facilitation [13,15-17]. For example, peripheral nerve injury produces profound changes in glial cells including morphological changes of microglia and astrocytes and increased expression of glial markers, such as CD11b, Iba-1 and GFAP [18]. Glia inhibitors or glia modifying drugs such as fluorocitrate and propentofylline can alter pain sensitivity [19-21]. While these early studies are important to demonstrate an overall role of glia in regulating pain sensitivity, they did not distinguish which type of glial cells is important in pain regulation.

Among three types of glial cells in the CNS, although oligodendrocytes and astrocytes are found in close apposition to neurons, microglia have gained more attention, in part because nerve injury-induced microglial changes are much more robust than that of oligodendrocytes and astrocytes. A recent microarray study shows that the most regulated genes following nerve injury are expressed in spinal microglia [22]. Nerve injury also induces a profound proliferation of spinal microglia [23]. Current studies on microglial regulation of pain are limited by the lack of specific markers for microglial activation. CD11b and Iba-1 are two of the most used markers for demonstrating microglial activation. However, whether CD11b and Iba-1 contribute to pain hypersensitivity remains unclear. Fortunately, nerve injury up regulates the ATP receptor P2X4 and the chemokine receptor CX3CR1 specifically in spinal microglia, and blocking these receptors results in decreased neuropathic pain [24-26]. The chemokine receptor CCR2 and Toll-like receptor-4 are also expressed in spinal microglia and contribute to neuropathic pain sensitization [27,28]. Different types of nerve injuries all induce complement cascade in spinal microglia, and mice null for C5 terminal complement component exhibit reduced neuropathic pain sensitivity [22]. Further, minocycline, a microglial inhibitor was shown to prevent or delay neuropathic pain [29,30]. In particular, intrathecal injection of ATP-activated microglia induces mechanical allodynia, indicating that microglial activation is sufficient to induce pain sensitization [24]. Activation of P2X4 receptor is likely to mediate neuropathic pain by producing BDNF in microglia [14].

### Activation of p38 MAPK in spinal microglia and neuropathic pain

The mitogen-activated protein kinases (MAPKs) are a family of evolutionally conserved molecules that play a critical role in cell signaling and gene expression. MAPK family includes three major members: extracellular signal-regulated kinase (ERK), p38, and c-Jun N-terminal kinase (JNK), representing three different signaling cascades. MAPKs are activated by phosphorylation and transduce a broad range of extracellular stimuli into diverse intracellular responses by both transcriptional and non-transcriptional regulation. p38 MAPK, which is activated by upstream kinase MKK3/MMK6, is regarded as a stress-induced kinase and plays a critical role in inflammatory responses. p38 inhibitor was shown to effectively alleviate rheumatoid arthritis and inflammatory pain [31-33].

**Figure 1:**
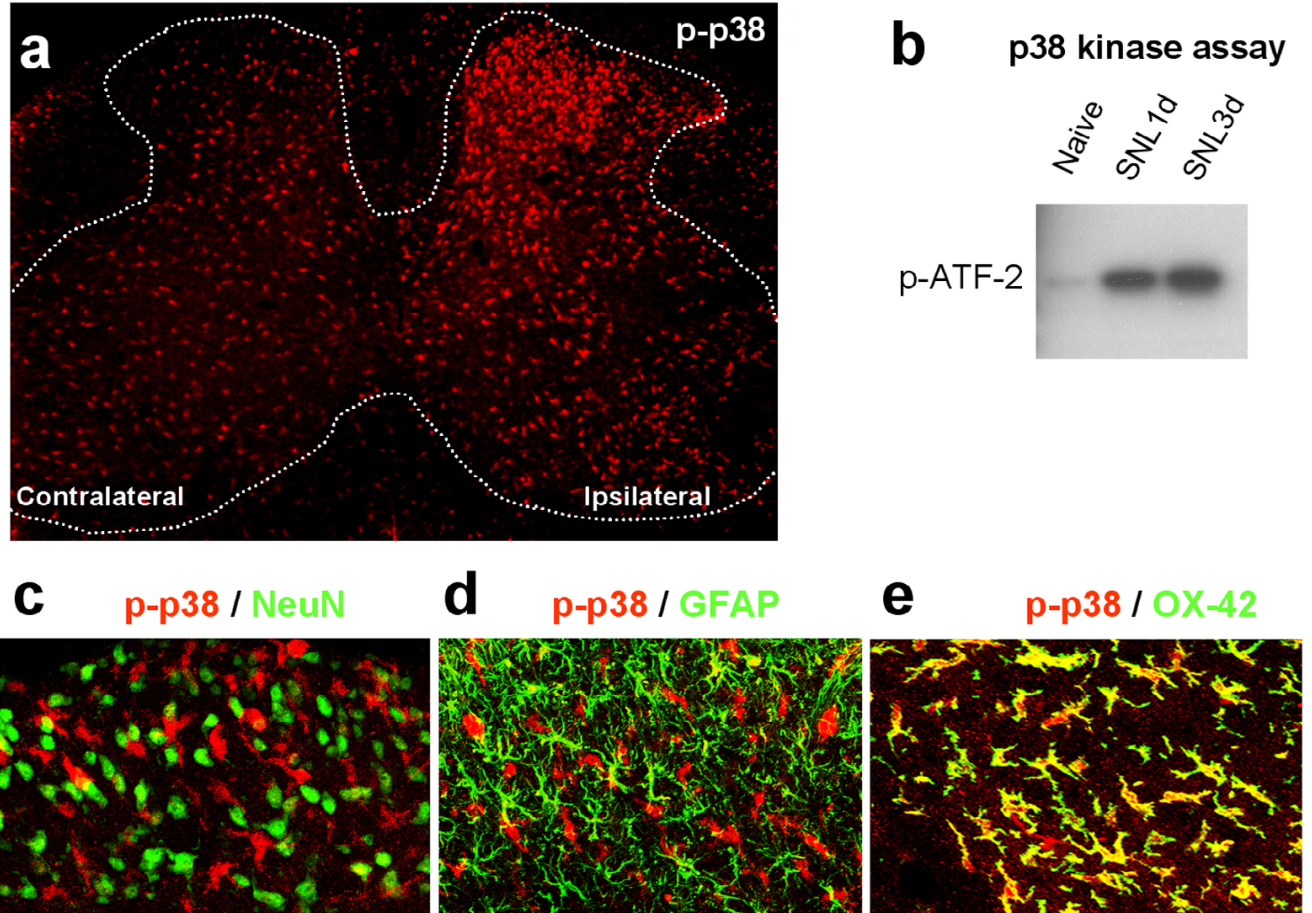
**Ligation of L5-spinal nerve (SNL) induces p38 activation in microglial cells in the spinal cord**. (a) Immunostaining of phosphorylated p38 (p-p38) in the spinal cord 3 days after SNL. Dotted line indicates the outline of the spinal cord gray matter. Modified from [34]. (b) p38 kinase assay reveals that SNL increases the phosphorylation of the p38 substrate ATF-2, suggesting an increase in p38 activity. To perform kinase assay, p-p38 was first immunoprecipitated with a p-p38 antibody, then ATF-2 fusion protein was added. Finally, pATF-2 level was detected using Western blotting with a pATF-2 antibody. (c-e) Double immunofluorescence indicates that p-p38 is not colocalized with NeuN, a neuronal marker (c) and GFAP, an astroglial marker (d), but colocalized with OX-42 (CD11b), a microglial marker (e). Modified from [34].

We used a phosphorylated p38 antibody to examine p38 activation in the rat spinal cord after spinal nerve ligation (SNL), a commonly used neuropathic pain model [34]. While phsopho-p38 (p-p38) levels are low in non-injured spinal cord, SNL induces a substantial increase in p-p38 levels in the injured side of the spinal cord (Fig 1a). The increase in p38 phosphorylation is accompanied by an increase in p38 activity, as shown by elevated phosphorylation of ATF-2, a substrate of p38 (Fig 1b). Unexpectedly, p38 is activated neither in NeuN-expressing neurons nor in GFAP-expressing astrocytes after SNL. Instead, p38 is activated in spinal cells labeled with microglial marker CD11b (recognized by OX-42 antibody, Fig 1c–e) [34]. p38 activation in spinal microglia was also reported in the SNL model by other investigators [35-37], as well as in the spared nerve injury model [38], after ventral root lesion [39] and spinal cord injury [40]. Apart from neuropathic pain conditions, microglial activation of p38 has also been shown in other pain conditions [41,42].

We have also examined p38 activation at different times following nerve injury. After SNL, p38 activation in spinal microglia begins on day one and peaks on day 3. It is noteworthy that the increase in p-p38 levels is maintained in spinal microglia even after 3 weeks, despite the fact that at this late time point p-p38 levels decline from the peak levels [43]. Although most studies confirm a specific microglial activation of p38 in the spinal cord, we do not exclude the possibility that moderate p38 activation may also be observed in other spinal cell types when different species or treatment conditions are tested.

### p38 inhibition and neuropathic pain

Mounting evidence indicates that p38 activation plays an important role in the development of neuropathic pain. Daily administration of the p38 inhibitor SB203580 intrathecally (i.t.) prevents SNL-induced mechanical allodynia [34,35]. p38 inhibitors FR167653 or CNI-1493 (i.t.) also prevents the development neuropathic pain symptoms in the spared nerve injury model [38] and in a sciatic inflammatory neuropathy model [44].

**Figure 2:**
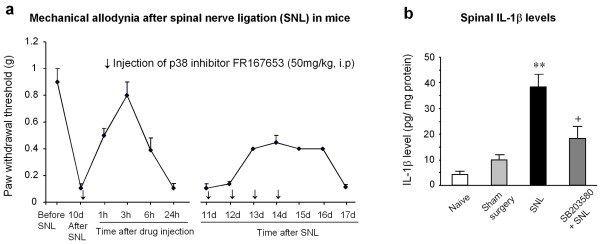
**(a) Reversal of tactile allodynia by p38 inhibition after spinal nerve ligation (SNL) on mice**. The p38 inhibitor FR167653 was injected (i.p., 50 mg/kg, in 10% DMSO) at 10 days after SNL. The same injection was repeated daily for additional 4 days (indicated with small arrows). SNL-induced mechanical allodynia was tested at 1 h, 3 h, 6 h, and 1 d after the first injection, then daily for another 6 days. Mean ± SEM (n = 5). Note that p38 inhibitor is still effective in reversing neuropathic pain. Interestingly, the effect is maintained for 2 more days after the last injection. **(b) Inhibition of SNL-induced IL-1β upregulation in the spinal cord by p38 inhibition**. ELISA shows that intrathecal injection of the p38 inhibitor SB203580 (10 μg, twice a day for 3 days) reduces SNL-induced IL-1β increase in the L5 spinal cord (dorsal part) 3 days after SNL (L5). **, P < 0.01, compared to Sham; ^+^, P < 0.05, compared to SNL, ANOVA.

Can p38 inhibitor or microglial inhibitor also reverse established neuropathic pain? This is an extremely important question regarding the role of p38 and microglia, and potential clinical treatment of neuropathic pain with p38 or microglia inhibitors. Early studies showed that SB203580 cannot reverse neuropathic pain symptoms when given after nerve injury ref36 taken away [45]. Similarly, a microglial inhibitor minocycline was shown to prevent/delay neuropathic pain, but not to reverse established neuropathic pain [29,30]. Minocycline also inhibits spinal p38 activation in a neuropathic pain condition [40]. These data suggest that p38 and microglia are only important for the induction or development of neuropathic pain, and p38 or microglia inhibitor can only be used to treat neuropathic pain at early stages, which would greatly limit the therapeutic potential of p38 and microglial inhibitors. However, further studies suggest that inhibition of p38 is also able to reverse neuropathic pain symptoms in different animal models [34,38,40,44,46]. Our data show that a single injection of the p38 inhibitor FR167653 (50 mg/kg, i.p.), 10 days after SNL, reverses mechanical allodynia for more than 6 hours, with a complete reversal at 3 hours. Further, multiple injections (once every day) of FR167653 persistently attenuate mechanical allodynia for several days, and pain attenuation is maintained for additional 2 days after the last injection (Fig 2a), supporting a role of p38 in the maintenance of neuropathic pain. Blockade of P2X4 signaling in microglia at 2 weeks after nerve injury can also reverse neuropathic pain [25]. Recently, a glial modifying drug propentofylline, given 6 weeks after nerve injury, was shown to reverse neuropathic pain by inhibiting microglial responses [47].

Nevertheless, the peak activation of p38 and microglia in the spinal cord occurs in the first several days of nerve injury, varying from 3 days to 2 weeks due to different injury conditions [43,48]. After its peak, p38 and microglial activation starts to decrease significantly, although still maintained at elevated levels at 3 weeks. In contrast, the peak activation of astrocytes in the spinal cord persists for a longer period [48]. Therefore, microglia are probably more important for the early development of neuropathic pain, although they are also involved in the maintenance of neuropathic pain.

### p38 isoforms

There are four different p38 isoforms: α, β, γ and δ. α and β are two of the major isoforms in the mature nervous system [31] and their activated forms are recognized by commercial p-p38 antibodies. While p38α is the most abundant isoform and expressed in DRG neurons (unpublished observation), p38β appears to be expressed in spinal microglia [49]. Current p38 inhibitors are not isoform-specific due to their design to target common-ATP binding sites on the kinases and structural similarities between the p38 isoforms. To explore the distinct role of p38 isoforms in the development of neuropathic pain, it is important to specifically inhibit a particular isoform. More importantly, isoform-specific inhibition will minimize the side effects produced by blocking all p38 isoforms with a general inhibitor. Interestingly, it has been shown that knockdown of p38β but not p38α with antisense oligonucleotides prevents acute pain sensitization [49]. However, the respective role of p38α and p38β in chronic pain conditions has not been examined. Also, therapeutic enthusiasm for antisense oligonucleotides is not high due to their low efficacy and specificity. A new strategy, such as RNA interference, may be helpful to develop potent and selective knock down of a specific isoform of p38.

### How does peripheral nerve injury activate p38 in spinal microglia?

Several studies began to explore the upstream mechanisms causing p38 activation in spinal microglia [26,36,50,51]. TNFα inhibitor prevents neuropathic pain development by inhibiting spinal p38 activation [36]. Spinal p38 is activated by intrathecal infusion of IL-1β and substance P, and p38 inhibitor abolishes the hyperalgesia produced by IL-1β and substance P [50,51]. In particular, recent studies have demonstrated that chemokines play important roles in mediating neural-glial interaction and neuropathic pain sensitization. Nerve injury induces an up-regulation of the chemokine MCP-1 in primary sensory neurons [48,52]. Interestingly, CCR2, one type of receptors for MCP-1, was expressed in spinal microglia; spinal p38 activation after nerve injury is reduced in CCR-2 null mice [27].

Unlike other chemokines that have multiple receptors, fractalkine (FKN), also called CX3CL1, only has one known receptor, CX3CR1. It is of great interest that the CX3CR1 receptor is expressed in spinal microglia and further upregulated after nerve injury [25,26]. On the other hand, FKN is primarily produced in DRG and dorsal horn neurons [25]. FKN has 2 forms, a membrane-bound form (large size) and a soluble form (small size) that mediate cell adhesion and chemotaxis, respectively. FKN is normally membrane-bound, but this membrane-bound FKN in the DRG is cleaved after nerve injury, leading to the release of FKN [26]. A recent study shows that cysteine protease cathepsin S (CatS), which is produced in microglia after nerve injury, can enhance neuropathic pain by cleavage of FKN on the membrane of DRG neurons. CatS-induced hyperalgesia is lost in CX3CR1 null mice [53]. FKN can also be cleaved by metalloproteinases (MMPs), such as MMP-9 that is released from DRG neurons after nerve injury (Xu and Ji, unpublished observation). Intrathecal infusion of FKN not only induces mechanical allodynia but also activates p38 in spinal microglia [26]. Perfusion of spinal cord slices with FKN activates p38 in microglia [26]. Conversely, a neutralizing antibody against CX3CR1 reduces both p38 activation and neuropathic pain [26]. Thus, FKN-CX3CR1-p38 cascade is required for the development of neuropathic pain.

After peripheral nerve injury, spontaneous activity (ectopic discharge), which is crucial for the genesis of neuropathic pain, can be recorded in axons as well as in DRG soma. Is this spontaneous activity required for p38 activation in spinal microglia? To produce long-term blockade of nerve activity, we applied bupivacaine microspheres to the sciatic nerve, which produces a persistent (> 1 week) conduction blockade due to slow release of bupivacaine [38]. Using this method for long-term blockade, we found that spontaneous activity is fully required to initiate p38 activation in spinal microglia. However, nerve conduction blockade does not reverse p38 activation when applied after nerve injury [38]. It is suggested that after initiation, microglia activation could be self-maintained given that multiple mediators released by microglia can also feed back to microglia via an autocrine or paracrine fashion. Since FKN released from neurons triggers microglial activation, it remains to be tested whether spontaneous activity after nerve injury can induce the cleavage of FKN.

### p38 and microglial signaling

How does p38 activation in spinal microglia result in pain hypersensitivity? As mentioned above, activated microglia produce numerous IFMs (e.g., IL-1β, IL-6, TNFα, PGE2, NO, BDNF), which are also produced in the spinal cord after nerve injury. All of these IFMs are implicated in pain facilitation [13-17,44,54,55].

p38 is known to regulate the synthesis of numerous IFMs via transcriptional regulation [31,32]. For example, SNL-induced IL-1β up-regulation is attenuated by spinal inhibition of p38 (Fig 2b). Especially, p38 has been shown to activate the transcription factor NF-κB in cultured microglia [56,57], leading to the expression of the IL-1β, IL-6, and COX-2 [58]. Interestingly, NF-κB is upregulated in spinal microglia after nerve injury (unpublished observation). Although it was traditionally believed that COX-1 is constitutively expressed while COX-2 is inducible, COX-1 was shown to be induced in spinal microglia after SNL [59] and COX-1 inhibitor can attenuate neuropathic pain [60]. It is worthy to investigate whether p38 is required for this upregulation. p38 activation after intrathecal IL-1β is required for the synthesis of iNOS [51]. In addition to p38 activation in spinal microglia, we found that ERK, another MAPK family member, is also activated in spinal microglia at early times (first several days) of neuropathic pain development, which is required for neuropathic pain sensitization [61]. p38 and ERK appear to be activated in different populations of spinal microglia. It is likely that the ERK and p38 pathways can act together to regulate neuropathic pain. For example, a combination of MEK and p38 inhibitors has been shown to suppress endotoxin-induced expression of iNOS and TNFα in microglia with a much greater efficacy than each inhibitor alone [62].

p38 can also regulate the synthesis of IFMs by posttranslational regulation. For example, p38 activates phospholipase A2 (PLA2) via its downstream kinase MAPKAP-2 (MAPK activated protein kinase-2). PLA2 plays an important role in inflammatory responses. The activation of PLA2 results in the genesis of arachidonic acid for prostaglandin production, which can be catalyzed via COX to produce PGE2 [32]. Several PLA2 forms are expressed in spinal cord, and inhibiting spinal PLA2 induces a potent antihyperalgesia [63]. PGE2 release evoked by intrathecal dynorphin requires spinal p38 activation [64]. p38 activation also causes a rapid release of IL-1β from spinal microglia. For example, following application of lipopolysaccharide to an ex vivo spinal slice preparation, there is a rapid activation of p38 in spinal microglia and a rapid secretion of IL-1β. Further, IL-1β secretion is prevented by p38 inhibitor [65].

### Concluding remarks and future directions

**Figure 3:**
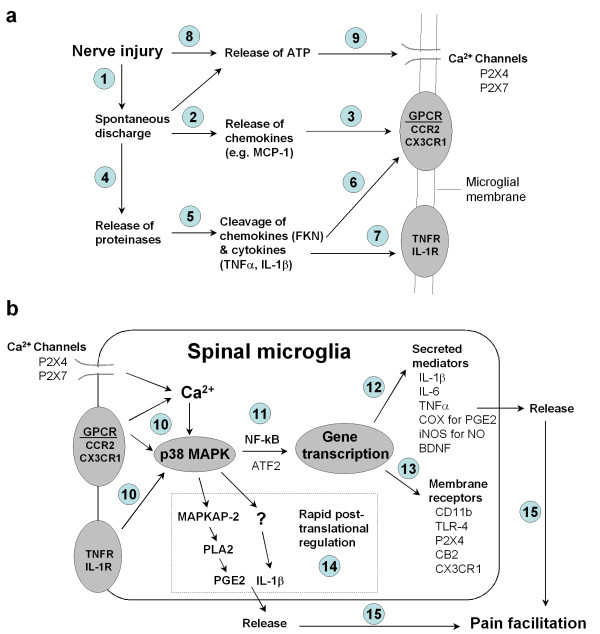
**Schematic representation of signal transduction in spinal microglia after nerve injury**. **(a) **Activation of microglial receptors after nerve injury. Peripheral nerve injury generates spontaneous activity (step-1) leading to the release of chemokines (e.g. MCP-1) from primary sensory DRG neurons (step-2). MCP-1 will activate CCR2 receptors on microglia (step-3). Spontaneous activity may also release the proteinases, leading to the cleavage of the chemokine FKN (step-5). After its cleavage from the membrane, FKN will be released to bind CX3CR1 receptor on microglia (step-6). Proteinases may also cleave the precursors of the cytokines TNFα and IL-1β, leading to the activation of TNFα and IL-1β receptors on microglia (step-7). Nerve injury will further release ATP (step-8), activating P2X4 and P2X7 receptors on microglia (step-9). **(b) **p38 activation in spinal microglia and downstream signaling of p38. Activation of GPCR, or cytokine receptors, or P2X receptors results in p38 MAPK activation in spinal microglia (step-10). p38 activation results in increased expression, through the transcription factor NF-κB (step-11) or other transcription factors (e.g. ATF-2), of secreted inflammatory mediators/growth factors (e.g., cytokines and BDNF, step-12) or of genes encoding membrane receptors (step-13). In addition, p38 also induces release of PGE2 and IL-1β via rapid posttranslational regulation (step-14). Upon release, these mediators will sensitize nociceptive dorsal horn neurons via presynaptic and postsynaptic mechanisms, leading to persistent pain hypersensitivity (step-15). Abbreviations used in Fig 3: BDNF, brain-derived neurotrophic factor; CatS, cysteine protease cathepsin S; DRG, dorsal root ganglion; FKN, fractalkine; GPCR, G-protein coupled receptor; IL-1β, interleukin-1beta; MAPK, mitogen-activated protein kinase; MAPKAP2: MAPK-activated protein kinase-2; PLA2, phospholipase A2; TNFα, tumor necrosis factor-alpha

In last several years there has been dramatically increasing interest in studying microglial regulation of neuropathic pain. We now know that many receptors such as P2X4, CX3CR1, and CCR2 are specifically expressed or up regulated in spinal microglia after nerve injury or spinal cord injury, which is essential for neuropathic pain development and maintenance. We also know that microglia enhance pain sensitivity by producing numerous inflammatory and pain mediators such as proinflammatory cytokines and BDNF. However, little is known as to how activation of these microglial receptors leads to the generation and release of inflammatory mediators. Many studies from different laboratories have shown that p38 MAPK is activated in spinal microglia and contributes to neuropathic pain development/maintenance. p38 stands in a central place in microglial signaling (Fig 3). First, activation of multiple microglial receptors can converge on p38 activation. Second, activation of p38 will lead to the synthesis and release of multiple inflammatory mediators. Therefore, understanding p38 signaling in microglia will greatly improve our knowledge of microglia-mediated pain regulation.

Currently, most people use CD11b or IBA-1 as markers for microglial activation. Although these are specific markers and show profound changes after injuries, their roles in neuropathic pain have not been clearly addressed, due to the lack of specific tools to block the action of these markers. In several cases, neuropathic pain is dissociated with CD11b expression [24,66]. Thus additional markers are needed to demonstrate microglia activation. Phosphorylated MAPKs (e.g. p-p38 or pERK) can be used as extra markers to reflect the activity of microglia after nerve injury, because phosphorylation is very rapid and sensitive in response to changes in cellular environment.

Can p38 inhibitors be used to treat clinical pain? Many pharmaceutical companies have been trying to develop specific p38 inhibitors. Several p38 MAPK inhibitors including SB281838, BIRB0796, Ro320-1195, and SCIO-469 have been tested in clinical trials for inflammatory diseases such as rheumatoid arthritis as well as for cancer [67,68]. Given that p38 is a critical contributor in several chronic pain conditions, p38 inhibitors should also be tested for clinical pain. A major concern is p38 inhibitors may produce psychiatric and cardiovascular side effects or liver toxicity. Development of more specific p38 inhibitors, such as activation status-specific inhibitors and isoform-specific inhibitors is highly demanded. Local delivery of p38 inhibitors or siRNA strategy may also be considered.

## Competing interests

The author(s) declare that they have no competing interests.
